# Plant cytoplasmic GAPDH: redox post-translational modifications and moonlighting properties

**DOI:** 10.3389/fpls.2013.00450

**Published:** 2013-11-12

**Authors:** Mirko Zaffagnini, Simona Fermani, Alex Costa, Stéphane D. Lemaire, Paolo Trost

**Affiliations:** ^1^Laboratory of Plant Redox Biology, Department of Pharmacy and Biotechnology, University of BolognaBologna, Italy; ^2^Department of Chemistry “G. Ciamician”, University of BolognaBologna, Italy; ^3^Department of Biosciences, University of MilanoMilano, Italy; ^4^Laboratoire de Biologie Moléculaire et Cellulaire des Eucaryotes, FRE3354, Institut de Biologie Physico-Chimique, Centre National de la Recherche Scientifique, Université Pierre et Marie CurieParis, France

**Keywords:** cysteine thiols, glycolytic glyceraldehyde-3-phosphate dehydrogenase, moonlighting protein, redox modifications, S-nitrosylation

## Abstract

Glyceraldehyde-3-phosphate dehydrogenase (GAPDH) is a ubiquitous enzyme involved in glycolysis and shown, particularly in animal cells, to play additional roles in several unrelated non-metabolic processes such as control of gene expression and apoptosis. This functional versatility is regulated, in part at least, by redox post-translational modifications that alter GAPDH catalytic activity and influence the subcellular localization of the enzyme. In spite of the well established moonlighting (multifunctional) properties of animal GAPDH, little is known about non-metabolic roles of GAPDH in plants. Plant cells contain several GAPDH isoforms with different catalytic and regulatory properties, located both in the cytoplasm and in plastids, and participating in glycolysis and the Calvin-Benson cycle. A general feature of all GAPDH proteins is the presence of an acidic catalytic cysteine in the active site that is overly sensitive to oxidative modifications, including glutathionylation and S-nitrosylation. In Arabidopsis, oxidatively modified cytoplasmic GAPDH has been successfully used as a tool to investigate the role of reduced glutathione, thioredoxins and glutaredoxins in the control of different types of redox post-translational modifications. Oxidative modifications inhibit GAPDH activity, but might enable additional functions in plant cells. Mounting evidence support the concept that plant cytoplasmic GAPDH may fulfill alternative, non-metabolic functions that are triggered by redox post-translational modifications of the protein under stress conditions. The aim of this review is to detail the molecular mechanisms underlying the redox regulation of plant cytoplasmic GAPDH in the light of its crystal structure, and to provide a brief inventory of the well known redox-dependent multi-facetted properties of animal GAPDH, together with the emerging roles of oxidatively modified GAPDH in stress signaling pathways in plants.

## INTRODUCTION

Glyceraldehyde-3-phosphate dehydrogenase (GAPDH) is a ubiquitous enzyme involved in glycolysis that catalyzes the conversion of glyceraldehyde-3-phosphate (G3P) to 1,3-bisphosphoglyceric acid (BPGA) in the presence of nicotinamide adenine dinucleotide (NAD^+^) and inorganic phosphate. Whereas animal cells contain only one isoform of GAPDH, plants contain several isoforms of GAPDH that are encoded by different types of genes (*gapA*, *gapB*, *gapC *and* gapCp*) and are located in different sub-cellular compartments. The products of *gapA* and *gapB* genes give rise to A_2_B_2_- and A_4_-GAPDH isozymes of chloroplasts that participate to the Calvin-Benson cycle (Michelet et al., 2013, submitted). Genes *gapC *and *gapCp *code for the subunits of glycolytic GAPDH isoforms, located either in the cytoplasm (GAPC) or in plastids (GAPCp; Petersen et al., 2003; Muñoz-Bertomeu et al., 2009, 2010). Depending in the species, each type of *gap *gene may be further duplicated, for example Arabidopsis contains two *gapA*, two *gapC* and two *gapCp* genes, but only a single *gapB*. All these GAPDH isoforms are defined phosphorylating, because they all catalyze *in vitro* the phosphorylation of the substrate G3P. However, phosphorylating GAPDHs of the Calvin-Benson cycle (A_2_B_2_- and A_4_-GAPDH) physiologically act in the opposite direction, and therefore catalyze the dephosphorylation of the substrate BPGA. In addition, the cytoplasm of plants also contains a non-phosphorylating GAPDH (GAPN) that oxidizes G3P directly to 3-phosphoglycerate and can bypass the GAPC-catalyzed reaction (Rius et al., 2006). GAPN belongs to the aldehyde dehydrogenase superfamily, and has no close functional/structural relationships with phosphorylating GAPDHs (Michels et al., 1994). Moreover, GAPN catalyses an NADP-specific irreversible reaction that represents an additional source of NADPH for the cytoplasm, whereas the reaction catalyzed by GAPC and GAPCp isozymes is reversible and strictly specific for NAD(H).

Glyceraldehyde-3-phosphate dehydrogenase, however, is not only an enzyme, but also a moonlighting protein. In animal cells, the concept of moonlighting proteins was introduced after the discovery of proteins that were able to perform additional functions in respect to their originally assigned ones (Jeffery, 1999; Kim and Dang, 2005). Often, additional and original functions were completely unrelated. A prototype of moonlighting proteins in animal cells is GAPDH that, besides acting as a glycolytic enzyme, is involved in several alternative functions including, among many others, DNA stability, control of gene expression and apoptosis (Sirover, 2012 and references therein). Several additional roles of GAPDH are linked to redox modifications of its catalytic cysteine that besides blocking its catalytic activity, have also profound effects on the capacity of GAPDH to interact with other proteins and eventually change its subcellular localization.

In contrast with the well established moonlighting properties of animal GAPDH, little is known on the multifunctional roles of plant cytoplasmic GAPDH, here referred as GAPC. Nonetheless, GAPC was identified as a potential target of diverse redox modifications and these modifications appear to be related to changes in subcellular localization in plant cells as well. Emerging evidence clearly show that, similar to animal GAPDH, plant cytoplasmic GAPDH may also perform alternative functions that are completely unrelated to its catalytic activity, strongly suggesting that plant GAPDH may also behave as a moonlighting protein.

The aim of this review is to provide an overview of what is known on plant cytoplasmic GAPDH, starting from its crystal structure and the structural features that determine its pronounced redox sensitivity. A comprehensive description of the molecular mechanisms underlying redox regulation of plant GAPCs will be provided, and a brief inventory of the redox-dependent multi-facetted properties of animal GAPDH will constitute a framework for the emerging role of oxidatively modified GAPDH in stress signaling pathways in plant cells.

## STRUCTURE AND REACTION MECHANISM OF GAPDH: AN ABUNDANT PROTEIN WITH A HIGHLY REACTIVE CATALYTIC CYSTEINE

All types of phosphorylating GAPDHs, both in plants and non-photosynthetic organisms, either involved in glycolysis or in the Calvin-Benson cycle, share a similar structure with variations on the theme. GAPDH is essentially a tetramer of identical or similar subunits, with the same overall organization in which the subunits are related to three symmetry axis. Subunits are identical in glycolytic GAPDHs (GAPC, GAPCp) and in A_4_-GAPDH of oxygenic phototrophs, and similar in the main photosynthetic isoform of land plants (A_2_B_2_-GAPDH). B-subunits share a high sequence homology with A-subunits but contain an extra sequence of 30 amino acids (C-terminal extension, CTE) that is essential for thioredoxin-based regulation and required for the association of A_2_B_2_-GAPDH isoforms into tetramers of tetramers (A_8_B_8_-GAPDH; Trost et al., 2006). In all types of GAPDH, each subunit contains the binding sites for the coenzyme (NAD(P)(H)) and for the substrates/products (G3P, P_i_, BPGA). This latter site hosts also the intermediates formed during the catalytic cycle via covalent modification of the active site cysteine.

Several structures of GAPDH isoforms from animal, microbial and plant sources are available (**Table 1**), although cytoplasmic GAPDH from rice (*Oryza sativa*; OsGAPC) is the only known crystal structure of a glycolytic GAPDH from a photosynthetic organism (PDB code 3E5R, Tien et al., 2012). Rice GAPC shares a very high sequence homology with GAPC from flowering plants, e.g., 85% identical to *A. thaliana* GAPC, and GAPDH genes of the GAPC-type are generally quite well conserved in eukaryotes and eubacteria (e.g., OsGAPC is 69% identical to *H. sapiens* GAPDH, and 50% identical to *B. stearothermophilus* GAPDH). Moreover, the crystal structure of OsGAPC can be nicely superimposed to known GAPDH structures of animals or eubacteria. Plastidic GAPCp forms are also quite similar in sequence to cytoplasmic counterparts from which they arose by gene duplication in early chloroplasts evolution (Petersen et al., 2003), but no crystal structure of GAPCp is yet available. Sequence identity decreases to about 40% when GAPC is compared to GAPA and GAPB subunits of chloroplastic GAPDH isozymes, but still the overall structure of these proteins remains closely related.

**Table 1:** List of selected GAPDH isoforms from diverse organisms for which the three-dimensional structure has been solved.

Pdb code	Organism	Nomenclature	Metabolic pathway	Reference
1GD1	*Bacillus stearothermophilus*	GAPDH	Glycolysis/gluconeogenesis	Skarzyñski et al. (1987)
1GPD	*Homarus americanus*	GAPDH	Glycolysis/gluconeogenesis	Moras et al. (1975)
1ZNQ	*Homo sapiens*	GAPDH	Glycolysis/gluconeogenesis	Ismail and Park (2005)
3E5R	*Oryza sativa*	GAPC	Glycolysis/gluconeogenesis	Tien et al. (2012)
1JN0	*Spinacia oleracia*	A_4_-GAPDH	Calvin-Benson cycle	Fermani et al. (2001)
2PKQ	*Spinacia oleracia*	A_2_B_2_-GAPDH	Calvin-Benson cycle	Fermani et al. (2007)
2EUH	*Streptococcus mutans*	GAPN	Glycolysis	Cobessi et al. (1999)

*Only GAPDH isoforms that are cited in the text are listed.*

**FIGURE 1:**
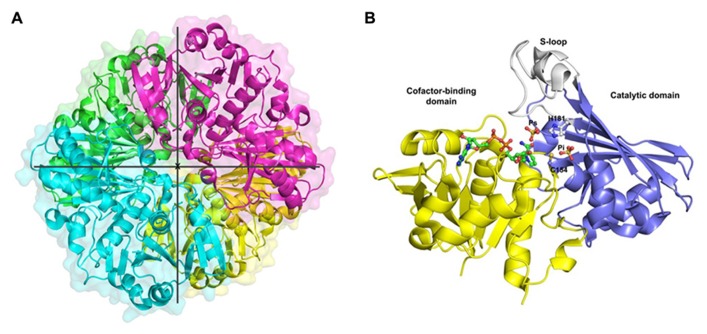
**Crystal structure of cytoplasmic GAPDH from rice.** **(A)** Ribbon representation of the homotetramer of *Oryza sativa* (Os) GAPDH (PDB code 3E5R), each subunit is differently colored. The three symmetry molecular axes are represented (the x-axis is perpendicular to the plane). **(B)** Ribbon representation of the crystal structure of a single OsGAPDH subunit. The cofactor-binding domain is yellow, the catalytic domain blue and the S-loop gray. The NAD^+^, the sulfate ions occupying the Ps and Pi sites and the catalytic residues (Cys-154 and His-181) are represented as ball-and-sticks.

This review is focused on GAPC-type GAPDH and a brief overview of the only known GAPC structure from plants is given. OsGAPC is a tetramer of four identical subunits related by three symmetry molecular axis (**Figure 1A**). The homotetramer is stabilized by several hydrogen bonds between monomers (or subunits). The monomer structure consists of two domains: a cofactor-binding domain comprising residues 1–153 and 318–337, and a catalytic domain composed of residues 154–317 (**Figure 1B**). The cofactor binding domain shows an α/β folding pattern typical of the Rossmann fold (Rossmann et al., 1975) with a central β-sheet composed of six parallel strands plus two extra antiparallel β-strands and five α-helices on both sides of the sheet. The additional segment of the NAD-domain (residues 318–337) folds in a long α-helix corresponding to the C-terminal region of the monomer. The catalytic domain is formed by three α-helices and eight β-strands and contains a long ordered loop called S-loop, stretching from residue 181 to 209, which extends toward the adjacent subunit in close proximity to the cofactor nicotinamide moiety (**Figure 1B**).

**FIGURE 2:**
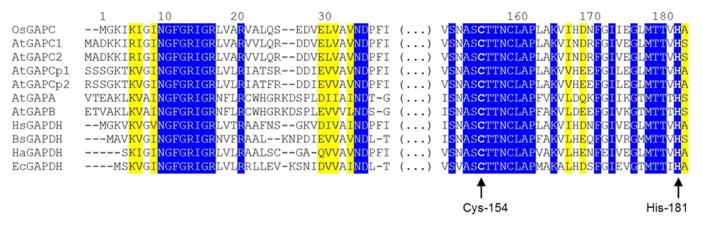
**Partial sequence alignment of GAPDH isoforms from different organisms.** The alignment is focused on two highly conserved regions of GAPDH, one belonging to the coenzyme-binding domain and one belonging to the catalytic domain. The first (N-terminal) region includes the strictly conserved sequence NGFGRIGR and other important residues for the binding of the pyridine nucleotide cofactor (Asp-35, Phe-37). The second region, located in the central part of GAPDH sequences, contains several residues involved in substrate binding including the catalytic cysteine (Cys-154), but also a second cysteine close to the active site (Cys-158) and His-181, essential for activating Cys-154. Residues are numbered according to the sequence of *Oryza sativa* (Os) cytoplasmic GAPDH (GAPC). Abbreviation and accession numbers: OsGAPC, *Oryza sativa* GAPC, Q0J8A4.1; AtGAPC1, *Arabidopsis thaliana* GAPC1, AEE74039.1; AtGAPC2, *Arabidopsis thaliana* GAPC1, AEE29016.1; AtGAPCp1, *Arabidopsis thaliana* GAPCp1, Q9SAJ6.1; AtGAPCp2, *Arabidopsis thaliana* GAPCp2, Q5E924.1; AtGAPA, *Arabidopsis thaliana* GAPA, AEE77191.1; AtGAPB, *Arabidopsis thaliana* GAPB; HsGAPDH, *Homo sapiens* GAPDH, P04406.3; BsGAPDH, *Bacillus stearothermophilus* GAPDH, PDB code 2DBV; HaGAPDH, *Homarus americanus* GAPDH, P00357; EcGAPDH, *Escherichia coli* GAPDH, ACI83895.1. Invariant residues are on a blue background while yellow background indicated residues with strongly similar properties. Catalytic Cys-154 (corresponding to Cys-149 and Cys-156 in BsGAPDH and AtGAPCs, respectively) and His-181 (corresponding to His-176 and His-183 in BsGAPDH and AtGAPCs, respectively) are highlighted by arrows. The sequences were aligned with the Clustal Omega program ().

A NAD^+^ molecule is bound to each enzyme subunit and anchored by several hydrogen bonds, either directly or through water molecules (Tien et al., 2012). In particular, the backbone atoms of the highly conserved N-terminal segment (Asn-9 to Arg-16: NGFGRIGR; **Figure 2**) interacts with the pyrophosphate moiety of the coenzyme, and the carboxyl group of Asp-35 (another strictly conserved residue of glycolytic GAPDHs, **Figure 2**) makes two hydrogen bonds with two adjacent hydroxyl groups (in position 2′ and 3′) of the ribose molecule linked to the adenine of NAD^+^. Other residues from the same subunit, and Asp-191 from the catalytic domain of the adjacent subunit further contribute to stabilize the cofactor. The correct holding of the adenine moiety of bound NAD^+^ is also determined by the conformation of Phe-37 side chain (conserved in glycolytic GAPDHs from higher plants and animals, but not in prokaryotes, **Figure 2**), that is 90°-rotated in apo-OsGAPC to allow the access of NAD^+^ to the coenzyme binding site (Tien et al., 2012).

**FIGURE 3:**
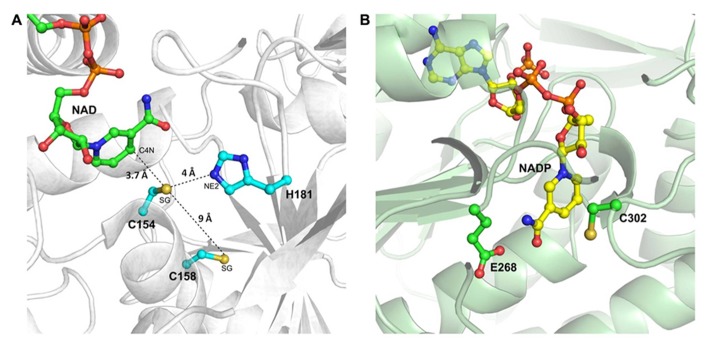
**The catalytic sites of rice cytoplasmic GAPDH and non-phosphorylating GAPDH from *Streptococcus mutants.*** **(A)** Magnified representation of OsGAPC catalytic site (PDB code 3E5R). Important catalytic residues, the cofactor NAD^+^ and residue Cys-158 are shown as ball-and-sticks. The distances of the sulfur atom (SG) of catalytic Cys-154 from the basic residue His-181 (atom NE2), the sulfur atom of Cys-158 and the cofactor (atom C4N) are indicated by dashed lines. **(B)** Catalytic site representation of *Streptococcus mutans* non-phosphorylating GAPDH (SmGAPN; PDB code 2EUH). The catalytic residues Cys-302 and Glu-268, and the cofactor NADP^+^ are in ball-and-sticks representation.

The structure of the active site of cytoplasmic OsGAPDH is very similar to that of other glycolytic and also chloroplastic GAPDHs. Sequence alignments show a highly conserved region between residues 150 and 161 (**Figure 2**), corresponding to the substrate binding site. The two most important catalytic residues are Cys-154 (corresponding to Cys-149 in *H. americanus* and *B. stearothermophilus* GAPDHs, the first solved GAPDH structures, **Table 1**) and His-181 (corresponding to His-176 in *H. americanus* and *B. stearothermophilus* GAPDHs; **Figure 2**). The side-chain of Cys-154 points toward His-181 and the distance between the sulfur atom of the first residue and the closest nitrogen atom (NE2) of the second residue is 4.03 Å on average (**Figure 3A**). The minimum distance of Cys-154 from the coenzyme nicotinamide ring is 3.69 Å (**Figure 3A**).

**FIGURE 4:**
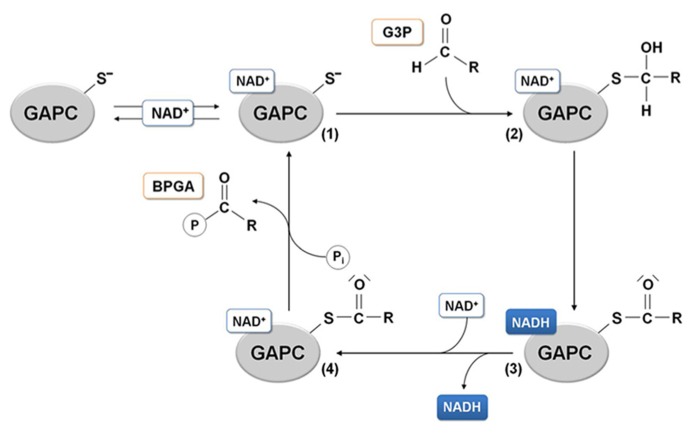
**GAPDH catalytic mechanism.** In the glycolytic direction, the binding of the substrate glyceraldehyde-3-phosphate (G3P) to the holo-enzyme containing NAD^+^ (1) leads to the formation of a hemithioacetal intermediate (2). In the next redox step, hydride transfer from the covalently bound substrate to the coenzyme allows formation of NADH, that can leave the acyl-enzyme (3) and be substituted by a molecule of NAD. The NAD-acyl-enzyme (4) can finally accept a free inorganic phosphate to release the product 1,3-bisphosphoglycerate (BPGA).

The presence of basic residues as histidines, arginines or lysines located in a close proximity with a cysteine residue generally at a distance lower or equal to 6 Å, enhances the deprotonation of the thiol (-SH) to the nucleophilic thiolate (-S^-^), making the cysteine quite reactive to oxidative modifications. The positioning of the catalytic cysteine at the N-terminal side of an α-helix (α6), might further increase its reactivity due to the helix overall dipole moment (Roos et al., 2013). According to these observations, it has been proposed that the histidine acts as an acid-base catalyst, extracting a proton from the catalytic cysteine and thus favoring a nucleophilic attack of the thiolate species on the substrate carbonyl group that generates a hemithioacetal intermediate (Moras et al., 1975). The subsequent oxidation step leads to the formation of the acyl-enzyme intermediate as a result of a hydride transfer (assisted by the nearby histidine) from the hemithioacetal to the nicotinamide ring of the coenzyme. NADH is then released and rapidly replaced by a new NAD^+^ molecule (**Figure 4**).

With the aim of evaluating the reactivity of the catalytic cysteine as a function of its chemical environment, the p*K*_a_ values for this residue were determined in GAPDHs from different sources. Values of p*K*_a_, estimated with different methods (cysteine titration with iodoacetamide or 2,2′-dipyridyl disulfide, or measurement of thiol ionization by UV absorbance), have been reported for pig liver GAPDH (5.5, Polgar, 1975), *E. coli* GAPDH (~5.7, Talfournier et al., 1998), *B. stearothermophilus* GAPDH (5.9, Talfournier et al., 1998), and *A. thaliana* GAPC (5.7, Bedhomme et al., 2012). The corresponding p*K*_a_ measured for the activating histidine (His-181) is around 8.0 (Talfournier et al., 1998). These values are very similar among different GAPDHs and reflect similar distances between the catalytic cysteine and the histidine, as observed in all GAPDH crystal structures available (e.g., 3.6 Å in *B. stearothermophilus* GAPDH and 3.4 Å in *A. thaliana* A_4_-GAPDH; average distance in different subunits). Overall, these values indicate that at physiological pH values the thiol group of the catalytic cysteine is deprotonated (i.e., thiolate, -S^-^) and involved in an ion pair with the protonated histidine. Additional features such as the positioning at the N-terminus of an α-helix, or the tridimensional arrangement of amino acids in the active site, further contribute to the acidic character of the catalytic cysteine.

In the OsGAPC structure, a second cysteine (Cys-158 corresponding to Cys-153 in *H. americanus* and *B. stearothermophilus* GAPDHs) is located, four residues away, in the same α-helix of the catalytic cysteine (**Figures 2, 3A**). The thiol groups of these two cysteines are oriented in opposite directions at a distance of about 9 Å, indicating that the formation of a disulfide bridge between these two cysteines, without significant distortion of the α-helix, is unlikely. Consistently, no disulfide bond was observed for *A. thaliana* GAPC1 under conditions promoting disulfide formation such as in the presence of oxidized thioredoxins (Bedhomme et al., 2012).

As already mentioned, plant cells contain a cytoplasmic non-phosphorylating NADP-GAPDH (GAPN), with low sequence homology with classical phosphorylating GAPDH (~20% with OsGAPC). The crystal structure of a GAPN from *Streptococcus mutans* (Sm) has been reported (**Table 1**; Cobessi et al., 1999). The catalytic mechanism comprises, similarly to GAPDHs, the formation of a covalent intermediate via the nucleophilic attack of the invariant Cys-302 on the aldhehydic function of G3P. However, the active site of SmGAPN has no basic amino acids with an equivalent role to the histidine found in GAPDHs (**Figure 3B**). Titration of Cys-302 in the apo-enzyme shows a p*K*_a_ of 8.5 that decreases to 6.1 upon NADP^+^ binding (Marchal and Branlant, 1999). Indeed, the binding of the coenzyme permits the chemical activation of Cys-302 and the formation of an efficient ternary complex intermediate (GAPN plus G3P and NADP^+^), through a local rearrangement of the active site and repositioning of the nicotinamide ring of the cofactor (D’Ambrosio et al., 2006). This suggests that GAPN uses a Cys activation mechanism alternative to the one observed for phosphorylating GAPDHs to achieve a similar catalytic pathway.

## PLANT GLYCOLYTIC GAPDH: A PERFECT PROTEIN TOOL TO INVESTIGATE REDOX POST-TRANSLATIONAL MODIFICATIONS

Reactive oxygen and nitrogen species (ROS and RNS, respectively) play an important role in redox signaling mainly through a set of reversible post-translational modifications (PTMs) of cysteine thiols on proteins (Hess et al., 2005; Besson-Bard et al., 2008; Dalle-Donne et al., 2009; Zaffagnini et al., 2012a).

The reaction between ROS and RNS with protein thiols mainly involves cysteine residues with unique physico-chemical properties. These residues are considered reactive when their ionization constant (p*K*_a_) is lower than that of free cysteines (8.3, Klomsiri et al., 2011), often ranging between 3 and 7. This implies that at physiological pH, these residues are predominantly found as thiolates (i.e., deprotonated, -S^-^), and thiolates are much stronger nucleophiles than thiol counterparts (-SH). Protein containing reactive cysteines can undergo different types of redox modifications in response to different ROS- and RNS-dependent redox signals. Physiologically, the most relevant ones are disulfide bond formation (S-S, intra- or inter-molecular), glutathionylation (S-SG), S-nitrosylation (S-NO) and oxidation to sulfenic acid (S-OH). Due to its unstable nature, sulfenic acids can generate disulfide bonds by reacting with a second cysteine nearby, or evolve to irreversible oxidized forms such as sulfinic and sulfonic acids (S-O_2_H and S-O_3_H, respectively). These redox PTMs are mostly controlled by oxidoreductases named thioredoxins (TRXs) and glutaredoxins (GRXs; Zaffagnini et al., 2012a).

Since 2001, numerous proteomic approaches have been developed to identify plant proteins putatively regulated by TRXs via dithiol/disulfide interchange or undergoing glutathionylation or S-nitrosylation (Besson-Bard et al., 2008; Lindahl et al., 2011; Zaffagnini et al., 2012a). Altogether, these studies allowed the identification of more than 500 putative targets involved in a wide array of cellular processes, including carbon metabolism, amino acid biosynthesis, redox control and stress responses (Lindahl et al., 2011; Michelet et al., this issue and references therein). However, compared to the large number of putative targets, very few studies reported a detailed biochemical investigation of the functional and structural effects of these redox modifications on each target protein and the molecular mechanisms involved.

In redox proteomic studies, plant glycolytic GAPDH (GAPC) has been identified as a potential target of TRX regulation (i.e., dithiol/disulfide interchange) and glutathionylation (Marchand et al., 2004; Dixon et al., 2005; Zaffagnini et al., 2012b). GAPC was also found to undergo S-nitrosylation in rice plants with increased NO levels (Lin et al., 2012) and in protein extracts from Arabidopsis leaves or cell suspensions treated with artificially released nitric oxide (NO; Lindermayr et al., 2005). In another study, GAPC from *A. thaliana* was identified as the prominent target of H_2_O_2_-dependent oxidation in plant protein extracts (Hancock et al., 2005).

The identification of plant GAPC as a target of multiple redox modifications is consistent with the fact that GAPC contains a reactive catalytic cysteine (referred in the following as Cys-149) with a low p*K*_a_ (e.g., 5.7 in *A. thaliana* GAPC1, Bedhomme et al., 2012; see previous section). At physiological pH, this residue is largely deprotonated (thiolate) and thus highly sensitive to ROS- and RNS-dependent oxidation with consequent inhibition of protein activity. Based on these considerations, the recombinant GAPC protein from *A. thaliana* has been extensively used as a protein tool to determine the molecular mechanisms underlying the different types of redox modifications and to investigate the enzymes and/or molecules involved in the control of these modifications.

### PLANT GAPC AS A TARGET OF PROTEIN SULFENATION

Protein sulfenation consists of the formation of a sulfenic acid after the reaction between a reactive cysteine and an oxidant molecule such as H_2_O_2_ through a two-electron oxidation process. The sensitivity of GAPC toward H_2_O_2_ has been tested by several research groups and in all cases a strong inhibition of enzyme activity was observed, suggesting that the highly reactive catalytic Cys-149 was indeed the target of H_2_O_2_ oxidation (Hancock et al., 2005; Bedhomme et al., 2012; Guo et al., 2012; Piattoni et al., 2013). The inhibitory effect of H_2_O_2_was also tested in the presence of GAPC substrates (BPGA in the reverse reaction and G3P in the forward reaction) and cofactor (NAD^+^). Whereas the cofactor is not able to protect GAPC from H_2_O_2_, a full protection of enzyme activity was observed in the presence of either substrates, consistent with the fact that either BPGA or G3P can covalently bind GAPC catalytic cysteine (Bedhomme et al., 2012; see **Figure 4**).

**FIGURE 5:**
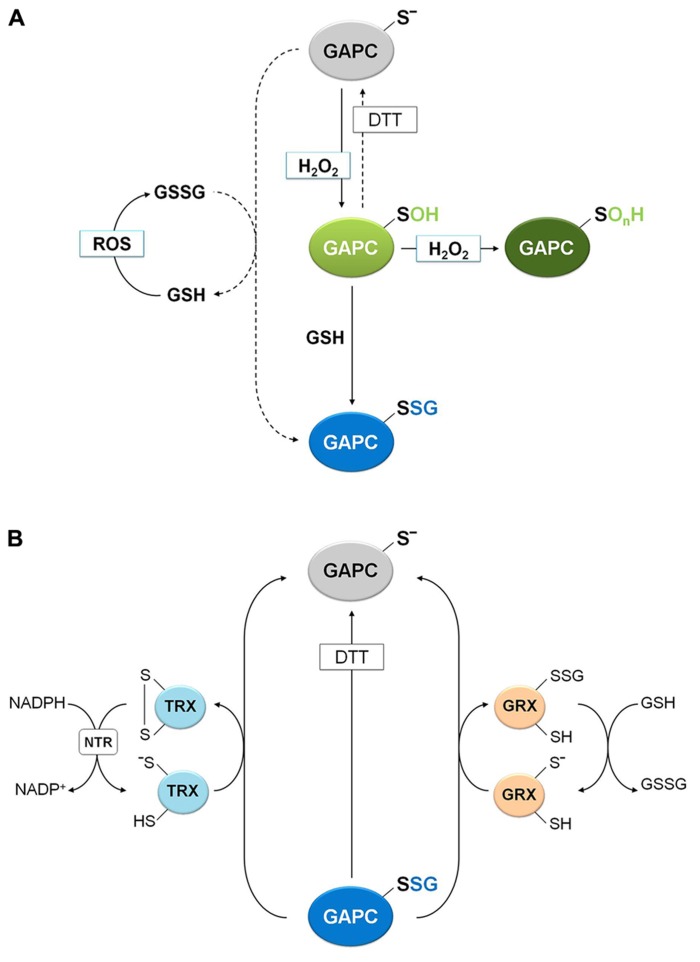
**Molecular mechanisms of plant GAPC glutathionylation and deglutathionylation.** **(A)** Oxidation and glutathionylation of plant GAPC. Plant GAPC can undergo primary oxidation to sulfenic acid (GAPC-SOH) in the presence of H_2_O_2_ that can subsequently react with GSH leading to the glutathionylated form (GAPC-SSG) and protecting the enzyme from irreversible oxidation (GAPC-SO_n_H). In principle, GAPC glutathionylation may also be performed by GSSG with release of GSH, but this reaction does not occur under physiological GSH/GSSG ratios (dashed arrow). GSSG can be formed by the reaction of GSH with ROS. **(B)** GRX- and TRX-dependent deglutathionylation mechanism of plant GAPC. The most reactive cysteine of both GRX and TRX (the N-terminal active-site cysteine) performs the nucleophilic attack on the glutathione-mixed disulfide on GAPC, resulting in the release of the reduced GAPC and the formation of a glutathionylated GRX intermediate (right side) or an oxidized TRX (left side). Subsequently, the mixed disulfide on GRX is reduced by a GSH molecule to form GSSG and reduced GRX and the oxidized TRX is reduced by NADPH-TRX reductase (NTR) in the presence of NADPH.

Beyond sulfenation, a modification that is fully DTT-reversible, cysteine thiolates may be further oxidized to sulfinic and sulfonic acids (Poole et al., 2004; Zaffagnini et al., 2012a). These oxidized forms are not reduced back by DTT and are generally considered irreversible although, in the case of 2-Cys-peroxiredoxins, the over-oxidation of the catalytic cysteine to sulfinic acid can be reversed by reduction by sulfiredoxins. Sulfiredoxins, first discovered in yeast (Biteau et al., 2003), are common to eukaryotes including plants (Rey et al., 2007). However, no other proteins than 2-Cys-peroxiredoxin have been shown to be reduced by sulfiredoxins. In the case of GAPC, the reversibility of H_2_O_2_treatments was examined after incubating with DTT or GSH, either soluble extracts from Arabidopsis suspension cells or the purified recombinant protein (Hancock et al., 2005; Bedhomme et al., 2012; Guo et al., 2012). Although a partial recovery of protein activity was observed after GSH/DTT treatment of soluble extracts, H_2_O_2_ induced a nearly irreversible inhibition of purified recombinant GAPC (Bedhomme et al., 2012; Guo et al., 2012), suggesting that Cys-149 sulfenate is unstable and can evolve into over-oxidized forms at rates that depend on experimental conditions (e.g., factors present in crude extracts, such as glutathione (GSH), may modulate over-oxidation; **Figure 5**).

### PLANT GAPC AS A TARGET OF PROTEIN GLUTATHIONYLATION

Protein glutathionylation, consisting in the formation of a mixed-disulfide between a protein thiol and a molecule of GSH, can act as a mechanism of protection against irreversible oxidation of reactive cysteine thiols but has also a role in modulating protein functions (Zaffagnini et al., 2012a). Although the precise mechanism leading to glutathionylation is still unclear *in vivo*, two major mechanisms may be envisioned: a GSSG-dependent thiol/disulfide exchange or a ROS-dependent sulfenic acid formation followed by reaction with reduced GSH. The role of glutathionylation for the protection of GAPC from over-oxidation was recently investigated (Bedhomme et al., 2012). The activity of GAPC was inhibited with similar kinetics by either H_2_O_2_ alone or by H_2_O_2_plus GSH, but only in the latter case DTT could totally recover the activity of the enzyme, indicating a reversible modification of catalytic Cys-149. A rational interpretation of this result is that GSH reacts with short-living Cys-149 sulfenates, generating a stable S-glutathionylated form that DTT can reduce, thus regenerating the protein thiol (**Figure 5**). Indeed, glutathionylation of GAPC by GSH plus H_2_O_2_ treatments was confirmed by MALDI-TOF mass spectrometry and immunodetection of biotin-labeled reduced GSH (BioGSH) (Bedhomme et al., 2012).

Glutathionylation of GAPC *in vitro* was also obtained with treatments with GSSG (Holtgrefe et al., 2008; Bedhomme et al., 2012). However, the effect on enzyme activity was limited because the *K*_ox_ of GAPC glutathionylation (i.e., the GSH/GSSG ratio at which 50% of the protein is glutathionylated) is very low (0.3 ± 0.05) compared with proteins that are easily glutathionylated by GSSG, like plastidial glutaredoxin S12 from poplar (*K*_ox_ = 300; Zaffagnini et al., 2012c). Therefore, the reaction of GAPC with GSSG might not have a significant role *in vivo*, since it requires GSH/GSSG ratios that are far lower than ever observed (Zaffagnini et al., 2012d and references therein). Moreover, the inhibitory effect of GSSG is completely blocked by either BPGA or NAD^+^ (Bedhomme et al., 2012). BPGA protection is in agreement with its covalent binding to Cys-149 (**Figure 4**), and is different from the effect of NAD^+^ that, although not linked to Cys-149, is close enough to shield Cys-149 from the attack of bulky GSSG, but not of smaller molecules like H_2_O_2_ and GSH. Overall, glutathionylation of GAPC is more likely to occur *in vivo* by reaction with both H_2_O_2_ and GSH, and can be considered either a mechanism of protection against over-oxidation or a mechanism of down-regulation of enzyme activity under oxidative stress conditions.

Removal of GSH from glutathionylated proteins (namely deglutathionylation) is, in most cases, a reaction that GSH cannot perform by itself with any significant efficiency. Glutathionylated GAPC (GAPC-SSG) and GRXs were recently used to investigate the molecular mechanisms of deglutathionylation reactions in a plant system (Bedhomme et al., 2012). Reactivation tests of GAPC-SSG were conducted in the presence of cytoplasmic class I GRXC1 from poplar, which contains a -CysGlyTrpCys- active site, and two GRXC1 mutants, each lacking one of the two active site cysteines (Bedhomme et al., 2012). These analyses revealed that GRXC1 efficiently catalyzes the deglutathionylation of GAPC-SSG following a monothiol mechanism that only requires the N-terminal active site cysteine (**Figure 5**). The first step of this mechanism consists in the nucleophilic attack performed by the most N-terminal active site cysteine of GRX on the mixed disulfide of GAPC-SSG. This reaction releases reduced, i.e., active, GAPC and a glutathionylated GRXC1 intermediate. In a second step, GSH removes the glutathionyl moiety from glutathionylated GRXC1, regenerating the reduced (i.e., active) GRXC1 (**Figure 5**). Similar results were described for other class I GRXs such as mammalian GRX1 and GRX2 (Gallogly et al., 2008), yeast GRX1 (Discola et al., 2009) and Arabidopsis GRXC5 (Couturier et al., 2011).

Glutathionylated GAPC was also used to investigate the deglutathionylating activity of different plant TRXs. These enzymes are known to control the activity of a large number of proteins through dithiol/disulfide exchange reactions (Lemaire et al., 2007; Schürmann and Buchanan, 2008; Lindahl et al., 2011), but recently, they have been proposed as important factors controlling the glutathionylation state of yeast proteins (Greetham et al., 2010; Tan et al., 2010). Interestingly, deglutathionylation of GAPC-SSG was found to be catalyzed by different plant cytoplasmic h-type TRX isoforms, although much less efficiently than GRXC1 (Bedhomme et al., 2012; **Figure 5**). TRXs contain two active site cysteine residues and the N-terminal one performs the nucleophilic attack on the mixed disulfide (**Figure 5**). In principle, the mechanism of TRX-mediated deglutathionylation could either involve an intermediate in which GAPC and TRX are disulfide-linked, or a glutathionylated TRX intermediate, as in the GRX-catalyzed mechanism. Although this point could not be resolved, in both cases TRX ends up with a disulfide that requires NADPH and NADPH-thioredoxin reductase (NTR) to regenerate the original thiols (**Figure 5**).

### PLANT GAPC AS A TARGET OF PROTEIN S-NITROSYLATION

**FIGURE 6:**
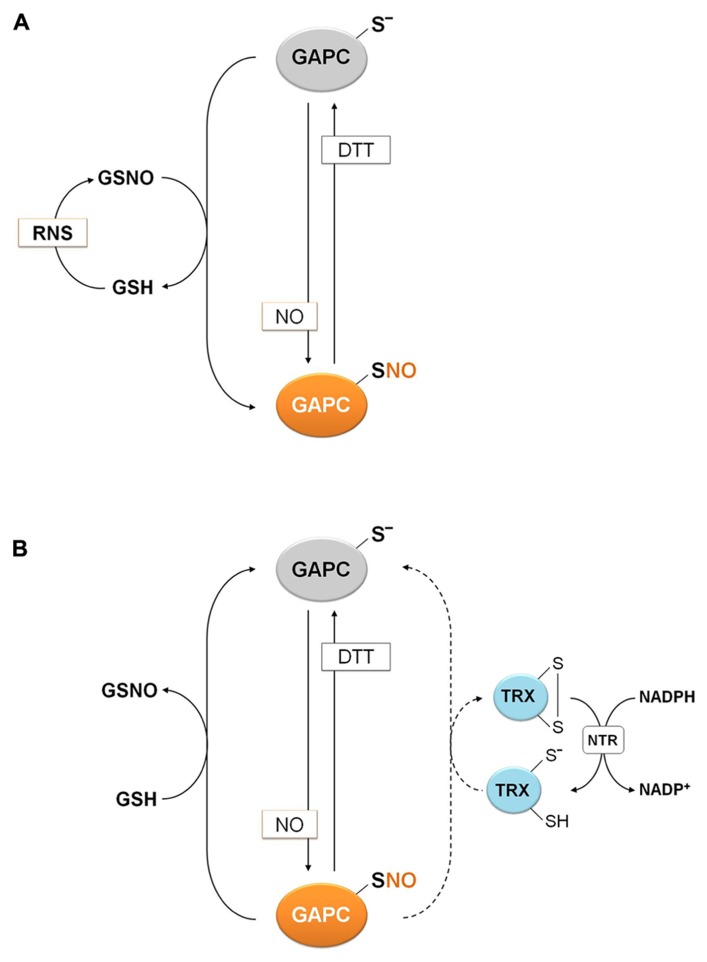
**Molecular mechanisms of plant GAPC nitrosylation and denitrosylation.** **(A)** Nitrosylation of plant GAPC. Plant GAPC undergoes reversible S-nitrosylation in the presence of NO donors or trans-nitrosylation by GSNO with concomitant release of GSH. GSNO can be generated by the reaction of GSH with RNS. **(B)** Denitrosylation mechanism of plant GAPC. Denitrosylation of plant nitrosylated GAPC (GAPC1-SNO) is not catalyzed by plant cytoplasmic TRXs (dashed arrow), but it is efficiently catalyzed by GSH with formation of GSNO.

Protein S-nitrosylation, consisting in the formation of nitrosothiols (S-NO) by reaction of protein thiols with NO, can be triggered chemically by RNS such as NO or by trans-nitrosylation reactions mediated by small nitrosothiols (e.g., nitrosoglutathione, GSNO) or by other S-nitrosylated proteins (Besson-Bard et al., 2008; Benhar et al., 2009; Astier et al., 2011; Yu et al., 2012). In the case of GAPC, NO-donors such as GSNO and sodium nitroprusside (SNP) were found to inhibit the NAD(H)-dependent GAPDH activity of total extracts of *A. thaliana*, and DTT was found to restore the activity almost completely (Lindermayr et al., 2005; Hancock et al., 2005). The mechanism of GSNO inhibition required further investigation since GSNO, besides acting as NO-donor, can also induce glutathionylation (Giustarini et al., 2005). Moreover, the inhibitory effect of SNP is controversial since the breakdown of this molecule (composed of a ferrous center surrounded by five tightly bound cyanide ligands and one linear NO ligand) releases one molecule of NO and five cyanide ions. Being cyanide reactive with protein thiols, it might contribute to inhibition. Recently, two papers addressed the question and demonstrated by the biotin switch method that GSNO induces S-nitrosylation of GAPC on its catalytic cysteine with concomitant inhibition of enzyme activity (Holtgrefe et al., 2008; Zaffagnini et al., 2013). Moreover, MALDI-TOF analysis demonstrated that S-nitrosylation of GAPC by GSNO largely prevailed over glutathionylation, both reactions being partially prevented by NAD^+^ (Zaffagnini et al., 2013). S-nitrosylation of GAPC was further confirmed by using a specific NO-donor such as DEA-NONOate (Zaffagnini et al., 2013), indicating that S-nitrosylation of GAPC may occur either through direct reaction with NO or GSNO-dependent trans-nitrosylation (**Figure 6**). The structural determinants that allow GSNO to act as a specific nitrosylating agent of GAPC remain to be understood, although the existence of specific binding motifs for GSNO, composed of acidic and hydrophobic residues has been proposed (Hess et al., 2005).

**FIGURE 7:**
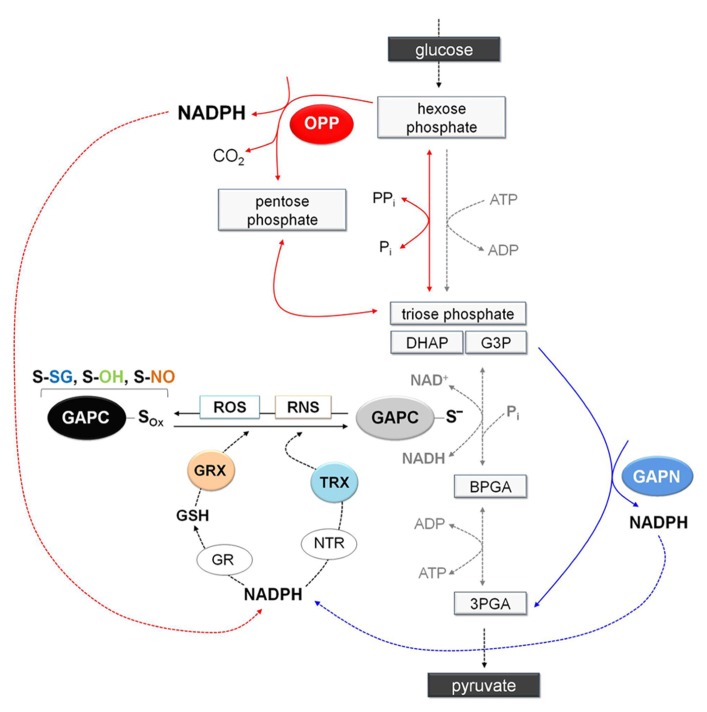
**Schematic representation of glycolysis showing the NADPH-producing systems in a situation of oxidative modification of GAPC.** Under stress conditions, GAPC might undergo different type of oxidative modifications (GAPC-S_ox_: sulfenation, S-OH; glutathionylation, S-SG; or nitrosylation, S-NO) with important effects on cytoplasmic primary metabolism. Indeed, inhibition of GAPC activity and the consequent down-regulation of glycolysis pathway would promote entry of glucose equivalents into the OPP pathway leading to the generation of NADPH (red arrows). Although inhibition of GAPC would down-regulate the glycolytic pathway, plant cells also contain a non-phosphorylating GAPDH (GAPN) that can by-pass the GAPC-catalyzed reaction providing an alternative source of NADPH for the antioxidant enzymes (blue arrows). Glutathione reductase and thioredoxin reductases (GR and NTR, respectively) are major antioxidants enzymes in the cytoplasm of plant cells. Glutathione reductase, using NAPDH as electron donor, can keep the glutathione pool reduced providing the reductant (GSH) for the efficient reduction of nitrosylated GAPC or the deglutathionylation via cytoplasmic glutaredoxins (GRXs). Alternatively, GAPC may be also deglutathionylated by a GSH-independent system involving NADPH, NTR and cytoplasmic thioredoxins (TRXs). Overall, redirection of primary metabolism in stressed plant cells would allow reinforcing the antioxidant systems and creating the conditions for recovery (e.g., reduction/reactivation of redox-modified proteins such as GAPC). 3PGA, 3-phosphoglycerate; BPGA, 1,3-bisphosphoglycerate; DHAP, dihydroxyacetone phosphate; G3P, glyceraldehyde-3-phospate.

The reduction of nitrosothiols on proteins, i.e., denitrosylation, entails two possible mechanisms, either dependent on reduced GSH or on the TRX system (NADPH, NADPH: TRX reductase and TRX; Benhar et al., 2009; Sengupta and Holmgren, 2011). The relative contribution of these two mechanisms was recently investigated using S-nitrosylated GAPC (GAPC-SNO) as a protein substrate (Zaffagnini et al., 2013). Cytoplasmic TRXs (h-type from either poplar or *Chlamydomonas reinhardtii*) were found to have little or no ability to denitrosylate GAPC-SNO in the presence of a complete TRX reducing system (TRXs plus NADPH and NTR b from *A. thaliana*; **Figure 7**). In contrast, GSH at physiological concentrations (2 mM) allowed a rapid and complete recovery of GAPDH activity, comparable with the reactivation kinetics observed with DTT. Interestingly, GSH-dependent denitrosylation activity was sensitive to the GSH/GSNO ratio but independent of the GSH/GSSG ratio, in agreement with GSNO being released in the denitrosylation reaction (Zaffagnini et al., 2013; **Figure 6**).

### POSSIBLE METABOLIC CONSEQUENCES OF PLANT GAPC OXIDATION

Since cytoplasmic GAPDH is sensitive to oxidative stress due to its reactive catalytic cysteine, oxidative modification of this residue invariably inhibits enzyme activity and, in animal cells at least, also glycolysis (Ralser et al., 2007). The inhibition of the glycolytic pathway might increase the flux of glucose through the oxidative pentose phosphate (OPP) pathway, a metabolic re-routing that ensures high NADPH/NADP^+^ ratios in the cytoplasm and is believed to play a fundamental role in counteracting the oxidative stress (**Figure 7**). While GAPDH-dependent down-regulation of the glycolytic pathway has been demonstrated in animal cells (Ralser et al., 2007), it remains to be demonstrated in plants, which contain non-phosphorylating GAPN in the cytoplasm. This enzyme generates NADPH in an irreversible reaction that could in principle bypass the GapC-catalyzed reaction (Rius et al., 2006). Furthermore, GAPN appears to be more resistant to H_2_O_2_-dependent oxidation/inactivation than plant GAPCs (Piattoni et al., 2013). While creating a potential bypass for the blocked glycolytic flux under oxidative stress, GAPN might also provide an alternative source of NADPH for the antioxidant enzymes (**Figure 7**).

## ANIMAL GAPDH: REDOX-DEPENDENT NON-GLYCOLYTIC (MOONLIGHTING) FUNCTIONS

Although mammalian GAPDH clearly retains its fundamental role as a glycolytic housekeeping enzyme in the cytoplasm, mounting evidence indicates that different redox PTMs can induce or regulate additional non-glycolytic functions of GAPDH. In this sense, GAPDH is considered as a moonlighting protein. Besides inhibiting enzyme activity, redox PTMs of GAPDH can induce or affect the interaction with other proteins or nucleic acids (Tristan et al., 2011; Sirover, 2011, 2012) or the covalent binding with its cofactor NAD (Mohr et al., 1996). Two major moonlighting functions of GAPDH in animal cells will be described. The first deals with the apoptotic cascade that is triggered by GAPDH S-nitrosylation; the second concerns the role of GAPDH in the post-transcriptional regulation of endothelin, a moonlighting function of GAPDH that is not induced, but regulated by redox PTMs.

### S-NITROSYLATION-DEPENDENT GAPDH ROLE IN APOPTOSIS

**FIGURE 8:**
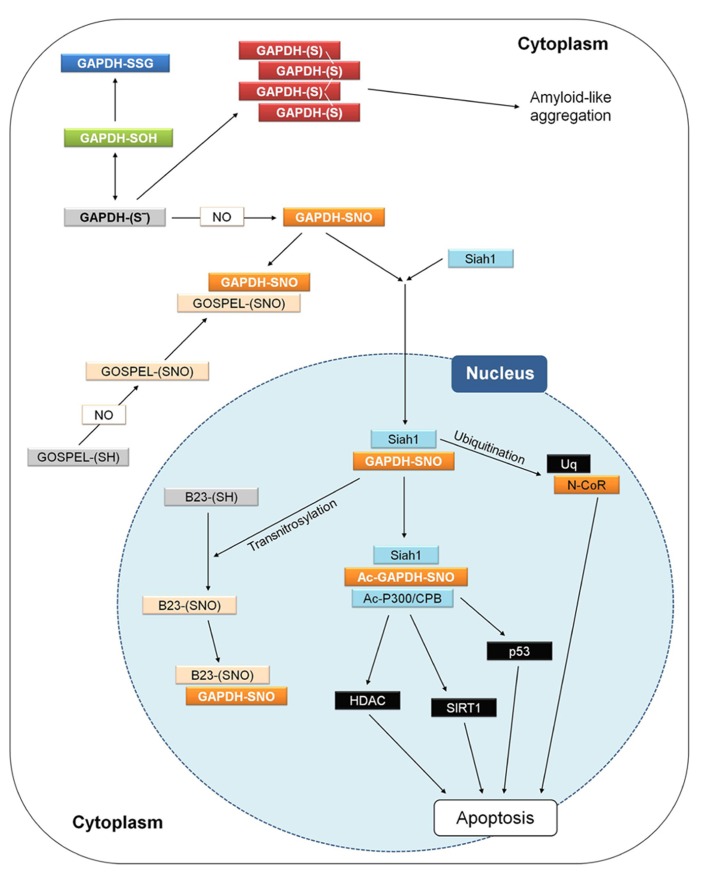
**GAPDH and apoptosis in animal cells.** Interactions and processes that have been observed in different studies and cell types are here summarized in a single hypothetical animal cell. Different apoptotic stimuli may induce NO biosynthesis and the figure shows how apoptosis in animal cells may be triggered by the initial nitrosylation of GAPDH, and counteracted by several processes. For a detailed explanation, see text (Animal GAPDH: redox-dependent non-glycolytic (moonlighting) functions). GOSPEL, GAPDH’s competitor Siah1 protein enhances life; N-CoR, nuclear co-repressor; Siah1, seven in absentia homologue 1.

Animal GAPDH is involved in the signaling cascade that governs NO-dependent apoptosis. After apoptotic stimuli, GAPDH is found in the nucleus in a number of cell systems, including neurons (Nakamura and Lipton, 2013). A fundamental prerequisite for GAPDH relocalization from the cytoplasm to the nucleus appears to be the S-nitrosylation of its catalytic cysteine. Diverse apoptotic stimuli induce NO biosynthesis in animal cells and S-nitrosylated GAPDH (SNO-GAPDH) acquires the ability to bind Siah1 (seven in absentia homologue 1), an E3-ubiquitin ligase with a nuclear localization signal (**Figure 8**). The SNO-GAPDH/Siah1 interaction is mediated by the C-terminal catalytic domain of GAPDH and has the effect of increasing the stability of Siah1. The complex SNO-GAPDH/Siah1 translocates to the nucleus and mediates cell death (**Figure 8**; Hara et al., 2005). Different mechanisms are involved in SNO-GAPDH/Siah1-mediated apoptosis (**Figure 8**). Diverse nuclear proteins are ubiquitinated by Siah1, including the nuclear co-repressor N-CoR (Hara et al., 2005), and SNO-GAPDH/Siah1 can also bind to acetyltransferase p300/CBP and in turn be acetylated on Lys-160. Interestingly, this interaction (in which Lys-160 of GAPDH plays an essential role) stimulates the autoacetylation of p300 and activates the acetylation of downstream targets like tumor suppressor p53 and others that, upon activation, induce cell death (Sen et al., 2008).

Siah1-mediated GAPDH translocation to the nucleus is counteracted by GOSPEL (GAPDH’s competitor Siah1 protein enhances life; **Figure 8**), a cytoplasmic protein that is more abundant in energy-demanding tissues in which GAPDH is also abundant. GOSPEL physiologically binds GAPDH and competes with Siah1 binding. GOSPEL is itself a target of S-nitrosylation (Cys-47) and this modification enhances GOSPEL ability to bind GAPDH. Under moderate stress conditions, GOSPEL efficiently protects GAPDH against the cytotoxic interaction with Siah1, but under harsher stress conditions the interaction of SNO-GAPDH with Siah1 prevails and apoptosis is consequently induced (Sen et al., 2009). Besides GOSPEL, other mechanisms can compete with Siah1-mediated nuclear translocation of GAPDH. Notably, the catalytic cysteine of GAPDH is physiologically acylated by its substrate G3P during the catalytic cycle (see Structure and Reaction Mechanism of GAPDH: an Abundant Protein with a Highly Reactive Catalytic Cysteine) and since acylated GAPDH cannot be S-nitrosylated no interaction with Siah1 can occur. Conditions of increased intracellular levels of G3P were reported to maintain cell survival by protecting GAPDH from S-nitrosylation (Lee et al., 2012). Actually, it may be speculated that any type of covalent modification of GAPDH catalytic cysteine, other than S-nitrosylation (e.g., glutathionylation), might in principle suppress the GAPDH-dependent apoptotic cascade.

Another mechanism counteracting the Siah1-mediated nuclear translocation of GAPDH is the formation of aggregates that immobilize GAPDH in the cytoplasm and may evolve into cytotoxic amyloid-like aggregates (**Figure 8**; Nakajima et al., 2007, 2009). In neurons, cytoplasmic GAPDH aggregates can form under oxidative stress conditions and are stabilized by intermolecular disulfides between catalytic cysteines and conformational changes of the protein with a concomitant enrichment of β-sheets (Nakajima et al., 2007, 2009). Formation of such abnormal GAPDH aggregates also promotes cell death, although through mechanisms independent of Siah1-dependent nuclear translocation.

The interaction between SNO-GAPDH and Siah1 can be counteracted also in the nucleus. This mechanism of regulation of the SNO-GAPDH/Siah1-dependent pro-apoptotic cascade depends on the ability of SNO-GAPDH to function as a trans-nitrosylase, i.e., to transfer its nitroso-group to a receiving thiol on a different protein (Kornberg et al., 2010; **Figure 8**). The neuroprotective nuclear protein B23/nucleophosmin is trans-nitrosylated by SNO-GAPDH and in its S-nitrosylated form binds to the SNO-GAPDH/Siah1 complex, thereby favoring the release of GAPDH from the complex and inhibiting the ubiquitinating activity of Siah1 (Lee et al., 2012). The neuroprotective role of B23 in the nucleus is reminiscent of that of GOSPEL in the cytoplasm: both proteins regulate cell survival by preventing SNO-GAPDH/Siah1 death-signaling under oxidative stress conditions.

The trans-nitrosylating activity of SNO-GAPDH in the nucleus is not restricted to B23. Other nuclear proteins that are similarly trans-nitrosylated include deacetylases such as SIRT1 and HDAC (Kornberg et al., 2010; **Figure 8**). However, S-nitrosylation of these targets is envisioned in the framework of normal processes of neuronal development rather than apoptosis and neurodegeneration (Nakamura and Lipton, 2013).

### GAPDH AND POST-TRANSCRIPTIONAL REGULATION OF GENE EXPRESSION

GAPDH can bind to RNA and several examples of post-transcriptional regulation mechanisms involving GAPDH can be found in the literature. Binding of GAPDH to messenger RNA may have contrasting effects: in some cases GAPDH increases mRNA stability (e.g., CSF1), in others stimulates mRNA degradation (ET-1) or causes inhibition of translation (AT1R; Sirover, 2011, 2012). In several systems, including plants, GAPDH was also found to bind viral RNA and strongly affect, either positively or negatively, viral replication (Prasanth et al., 2011; see Plant Glycolytic GAPDH: Metabolic and Non-Metabolic Functions). At least in the case of human endothelin (ET-1) discussed below, binding of GAPDH to mRNA was experimentally demonstrated to be redox-regulated (i.e., inhibited by redox PTMs of GAPDH itself), but there are reasons to believe that redox PTMs may have a more general effect on GAPDH-RNA interactions.

Post-transcriptional regulation of the vaso-constricting peptide endothelin (ET-1) is based on the ability of human GAPDH to bind AU-rich elements (ARE) of mRNA. AREs are common cis-regulatory elements of 3′-untranslated regions (UTR) of eukaryotic messenger RNAs and are important determinants of mRNA stability in mammals. Proteins binding to these elements can either increase or decrease mRNA stability and thus affect protein expression levels (Ahmed et al., 2011). The mRNA of the endothelin ET-1 contains in its 3′-UTR an ARE forming a secondary structure. By binding to this ARE, GAPDH causes mRNA unwinding and favors a faster degradation through ribonucleases. The mRNA binding activity of GAPDH occurs in its nucleotide binding domain and competes with the binding of NAD^+^ required for enzymatic activity (Nagy and Rigby, 1995). Interestingly, GAPDH-mRNA binding activity also depends on glycolytic GAPDH catalytic cysteine (the catalytic cysteine mutant does not bind ET-1 mRNA) and is prevented by treatments with GSSG or GSNO, suggesting that conditions leading to oxidative modifications of GAPDH (see Plant Glycolytic GAPDH (GAPC): a Perfect Protein Tool to Investigate Redox Post-Translational Modifications) may contribute to ET-1 post-transcriptional regulation *in vivo* (Rodríguez-Pascual et al., 2008).

Thanks to its capability to interact with AREs via its Rossmann fold, GAPDH was shown to bind many other types of mRNA, and may well exert a more general role in post-transcriptional regulation. Although only in the case of ET-1, GAPDH-mRNA interaction was experimentally shown to be redox-regulated, it is plausible that this property may also apply to other GAPDH-mRNA combinations, as long as different mRNAs may bind to GAPDH in a similar fashion.

## PLANT GLYCOLYTIC GAPDH: METABOLIC AND NON-METABOLIC FUNCTIONS

Compared to mammalian cells in which a single gene codes for GAPDH, Arabidopsis contains two cytoplasmic isozymes, GAPC1 (At3g04120) and GAPC2 (At1g13440), 98% identical in amino acid sequence, and both similarly contributing to the total NAD-specific GAPDH activity of the cytoplasm (Guo et al., 2012; Vescovi et al., 2013). The presence of more than one GAPC isoform is common in higher plants.

### METABOLIC ROLE OF GLYCOLYTIC GAPDH IN PLANTS

In spite of its role in a fundamental metabolic pathway such as glycolysis, reduction of GAPC protein levels in transgenic plants generally leads to relatively mild phenotypes under normal growth conditions, in agreement with the notion that plants contain possible alternatives to cytoplasmic GAPC, e.g., non-phosphorylating GAPN and plastidic GAPDHs (A_4_- and A_2_B_2_-GAPDH and GAPCp).

Partial suppression by antisense technology of cytoplasmic GAPC was obtained in transgenic potato (Hajirezaei et al., 2006). In these plants, NAD(H)-dependent GAPDH activity was reduced in both leaves, stems and tubers, but no clear phenotypic alterations were detected. In Arabidopsis, delayed growth, reduction of fertility and alterations of seed and fruit development were instead observed in both knock-out and antisense mutants of GAPC1. These traits were associated with a reduced respiratory rate, suggesting a metabolic-related phenotype (Rius et al., 2006). However, in another study, an Arabidopsis double mutant lacking both GAPCs (Guo et al., 2012) showed no particular phenotypic alterations under normal growth conditions, though defects in stomatal regulation made these plants overly sensitive to water stress. This effect was elegantly explained by a proposed role of GAPC1/GAPC2 in sensing H_2_O_2_ and promoting phosphatidic acid (PA) production in guard cells (Guo et al., 2012). As discussed below this function of GAPC does not rely on GAPDH enzymatic activity and is actually a moonlighting activity of the plant enzyme.

Quite unexpectedly, a strong metabolic-related phenotype is displayed by GAPCp double mutants in Arabidopsis. These plants are dwarf and suffer of arrested root development and sterility. In spite of the little contribution, in quantitative terms, of GAPCp to the total glycolytic activity of plant tissues, GAPCp (together with phosphoglycerate kinase) is essential in providing 3-phosphoglyceric acid (3-PGA) precursors for serine biosynthesis in plastids through the phosphorylating pathway (Ho and Saito, 2001). Lack of serine in non-photosynthetic tissues was shown to be the major reason for root growth inhibition, but also for defects in pollen and embryo development (Muñoz-Bertomeu et al., 2009, 2010).

### NON-METABOLIC ROLES OF PLANT GLYCOLYTIC GAPDH

**FIGURE 9:**
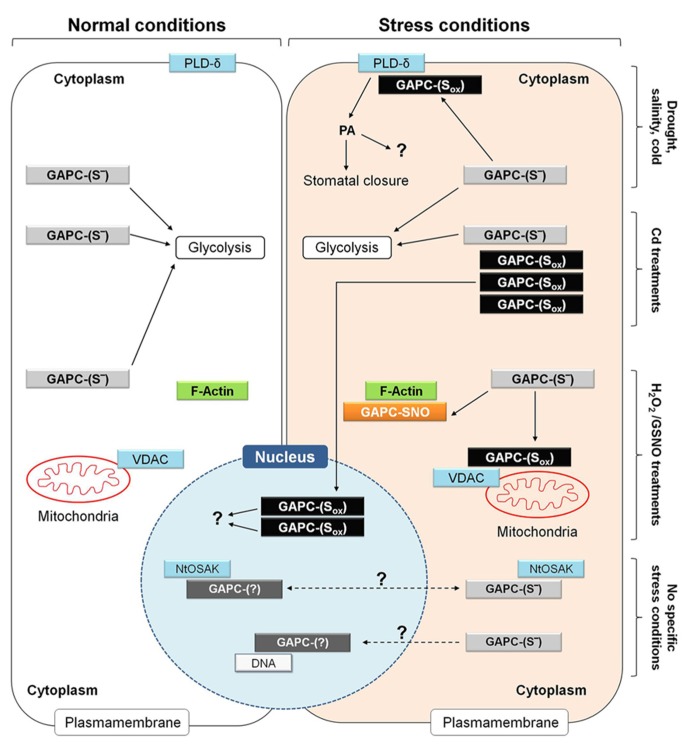
**Metabolic and non-metabolic functions of GAPC in plant cells under normal and different stress conditions.** Functional roles of GAPC that have been observed in different studies and cell types are here summarized in a single hypothetical plant cell. Under normal conditions, GAPC fulfills metabolic function by participating to glycolysis (left side of the figure) while it can play non-metabolic functions under specific stress conditions (e.g., drought, salinity and cold stress, treatments in the presence of cadmium (Cd), hydrogen peroxide (H_2_O_2_) and nitrosoglutathione (GSNO; right side of the figure). Overall, these conditions are supposed to induce different type of redox modifications of GAPC (GAPC-S_ox_) and stimulate interaction with several partner proteins or to induce protein expression, accumulation of inactive protein and relocalization to the nucleus. Additional functions of GAPC are not clearly related to either specific stress conditions or redox modification or sub-cellular localization of the GAPC protein and are indicated by dashed arrows. For a detailed explanation, see text (Plant Glycolytic GAPDH: Metabolic and Non-Metabolic Functions). NtOSAK, *Nicotiana tabacum* osmotic stress activated kinase; PA, phosphatidic acid; PLD-δ, phospholipase D-δ; VDAC, voltage-dependent anion channels.

Besides the multitude of examples in mammalian cells, emerging evidence for non-metabolic functions of glycolytic GAPDHs (GAPCs) are also coming from research carried out in plants (**Figure 9**). Here we will present some recent discoveries in which plant GAPCs have started to reveal their functional plasticity.

Arabidopsis mutants in which both GAPC isoforms were knocked out are impaired in ABA-induced stomatal closure, and consequently suffer of a higher transpirational water loss than wild type plants under drought stress (Guo et al., 2012). Drought stress and ABA are known to induce ROS production by NADPH oxidase in guard cells and GAPC is an established target of H_2_O_2_ (Hancock et al., 2005), probably also in guard cells. It was demonstrated in Arabidopsis leaves, that oxidation of GAPCs inhibits enzyme activity but stimulates the physical interaction between GAPCs and phospholipase D-δ (PLD-δ), which is then activated (**Figure 9**). PLD-δ is bound to the plasma membrane and once activated releases PA from phospholipids, with preference for phosphatidylethanolamine. Synthesis of PA in plants is triggered in response to various biotic and abiotic stresses, including pathogen infection, drought, salinity, wounding and cold, and is performed by many different PLD-isoforms (Testerink and Munnik, 2005). However, oxidized GAPC interacts specifically with PLD-δ, and PA synthesized by PLD-δ appears to induce stomatal closure through activation of mitogen-activated protein kinases, though other mechanisms may also be operative. Intriguingly, GAPC itself is also a target of PA (McLoughlin et al., 2013) and GAPC2 undergoes a proteolytic cleavage after binding to PA *in vivo *(Kim et al., 2013). Although the function of this process is not clear yet, it may represent a feed-back mechanism to down-regulate the GAPC-mediated PA-signaling (Guo et al., 2012). In any case, the activation of PLD-δ by oxidized GAPC is a clear example of a non-metabolic function of GAPDH in plants.

Similar to mammalian cells, also in plant cells GAPDH was detected both in the cytoplasm and in the nucleus (Bae et al., 2003; Anderson et al., 2004; Holtgrefe et al., 2008; Waver et al., 2010; Kim et al., 2013; Vescovi et al., 2013). The nuclear localization of a glycolytic enzyme is a good hint for a moonlighting function, but at difference with mammalian GAPDH for which the mechanism of nuclear translocation and the function in apoptosis has been well established (Hara et al., 2005; **Figure 8**), studies on nuclear GAPDH in plants are still in their infancy.

In a recent study (Vescovi et al., 2013), cadmium (Cd) treatments of Arabidopsis seedlings were found to induce the translocation of GAPC1-YFP chimeric protein from the cytoplasm to the nucleus in root tip cells (**Figure 9**). In these experiments, the GAPC1-YFP chimeric protein was expressed under the control of the GAPC1 endogenous promoter. This proved essential to avoid artifacts, as stronger constitutive promoters (e.g., CaMV-35S) may cause nuclear localization of GAPC as a mere consequence of its extremely high intracellular concentration (Vescovi et al., 2013). In Arabidopsis plants transformed with the endogenous GAPC1 promoter, translocation and accumulation of GAPC-YFP in the nucleus was thus a genuine effect of Cd treatments of Arabidopsis roots, that was found to correlate, in the same cells, with NO accumulation and oxidation of the GSH pool, as detected by the redox probe roGFP2. Inhibitors of GSH biosynthesis or catalase inhibitors, both treatments causing cytoplasmic oxidation, induced GAPC1 protein accumulation in a similar way as Cd treatments, suggesting that GAPC1 promoter activation may be mediated by unknown oxidized elements of the cytoplasm, rather than Cd itself (Vescovi et al., 2013). Independent works have also reported the up-regulation of GAPC (particularly GAPC1) under other stress conditions, including salt stress (Jiang et al., 2007), and oxidative stress by menadione (Lehmann et al., 2009). Interestingly, in the case of Cd-treated Arabidopsis roots, nuclear-accumulated GAPC1 was apparently inactive as an enzyme (**Figure 9**), consistent with the high sensitivity of GAPC to inactivation by oxidants like NO, H_2_O_2_, GSNO or GSSG (Lindermayr et al., 2005; Holtgrefe et al., 2008; Bedhomme et al., 2012; Guo et al., 2012; Vescovi et al., 2013; Zaffagnini et al., 2013). Therefore, it is concluded that Cd treatments may impose cellular oxidized conditions that, on one side stimulate GAPC1 gene expression, but on the other favor its inactivation and nuclear translocation (**Figure 9**).

Cadmium-induced nuclear translocation of GAPC1 in Arabidopsis reminds the nuclear translocation of mammalian GAPDH upon apoptotic stimuli. However, in this latter case translocation is triggered by the S-nitrosylation of GAPDH catalytic cysteine and inhibited by its mutation to serine [Hara et al., 2005; see Animal GAPDH: Redox-Dependent Non-Glycolytic (Moonlighting) Functions], while in Cd-treated Arabidopsis the nuclear relocalization of GAPC1 was stimulated by the substitution of the catalytic cysteine with a serine (Vescovi et al., 2013). This result seemed to rule out the involvement of a redox modification of the catalytic cysteine for the nuclear translocation of GAPDH in Arabidopsis. Recently, a mechanism based on the acetylation of three lysine residues was alternatively proposed for the nuclear translocation of GAPDH after the apoptotic stimulus in animal cells (Ventura et al., 2010). Whether a mechanism of this type may apply to Cd-induced nuclear translocation of GAPC in plants is still an open question.

In BY-2 tobacco cells, cytoplasmic GAPDH isoforms NtGAPCa and NtGAPCb were found to interact, both in the cytoplasm and the nucleus, with an osmotic stress activated kinase (NtOSAK; **Figure 9**), belonging to the SnRK2 family (sucrose non-fermenting 1-related protein kinase 2, Waver et al., 2010). This kinase is activated by phosphorylation under osmotic stress and NO treatments, and by the interaction with GAPC isoforms that were found partially S-nitrosylated under these conditions. Interestingly, GAPC mutants with no cysteines in the active site (because converted to serines) interacted with NtOSAK only in the cytoplasm and no complexes were detected in the nucleus, suggesting that GAPC S-nitrosylation was required for either nuclear translocation of GAPC itself or for the interaction with NtOSAK within the nucleus. Anyhow, GAPC/NtOSAK complexes were neither stimulated by salt stress nor by NO-donors, and therefore the function of the whole system still remains obscure (**Figure 9**).

Whatever the mechanism required for nuclear localization, a clear role of plant GAPDHs in the nucleus is still missing. Preliminary evidence that GAPC may bind to a DNA sequence coding for NADP-malate dehydrogenase (Holtgrefe et al., 2008; **Figure 9**) confirmed the results of a previous yeast one-hybrid screen (Hameister et al., 2007), but the effect of such interaction is unknown. An interesting, alternative scenario for the role of nuclear GAPC might come from the demonstration that mammalian and also chloroplastic GAPDHs display uracil glycosylase activity (Wang et al., 1999; Mazzola and Sirover, 2003). The uracil glycosylase activity of mammalian GAPDH is associated with its monomeric form present in the nucleus (Mazzola and Sirover, 2003). Plant cytoplasmic GAPDH has not yet been tested in this regard. However, bearing in mind that the uracil glycosylase activity is important for the maintenance of DNA integrity and preventing mutagenesis by eliminating uracil from DNA molecules, it is possible that the GAPC nuclear accumulation under oxidative stress in plants may play a role in DNA protection. In support of this hypothesis, Cd, a major inducer of GAPC nuclear translocation, has been reported to induce not only oxidative stress (De Michele et al., 2009; Vescovi et al., 2013), but also DNA damage and mutations in Arabidopsis plantlets (Liu et al., 2012).

The capability of GAPDH to bind nucleic acids does also explains its recently discovered role in regulating virus replication in plants. Indeed GAPC interacts with the replicase complex of different phytopathogenic viruses with a single-stranded, positive-sense RNA genome. The effect of GAPC binding consists in either repressing or stimulating virus replication. In *Nicotiana benthamiana* infected with the Bamboo Mosaic Virus (BaMV), GAPC binds the 3′-untranslated region of both BaMV genomic and satellite RNA and negatively regulates virus replication. Consistently, in GAPC-overexpressing plants, BaMV replication is further inhibited, whereas GAPC-silenced plants are more susceptible to the virus (Prasanth et al., 2011). For Tomato Bush Stunt Virus (TBSV), again infecting *Nicotiana benthamiana*, the effect of GAPC was opposite. In this case, GAPC is incorporated into the viral replicase complex and binds to the 3′-UTR of the negative-sense RNA of the virus, in correspondence of an AU-pentamer. This binding favors retention of the (-)RNA by the replication complex, such that synthesis of positive strands are favored. Moreover, by binding the viral polymerase subunit p92^pol^, GAPC help recruiting the RNA-dependent RNA-polymerase on the negative strand, again favoring positive RNA strands production. Although the N-terminal part of GAPC, including the catalytic cysteine, is required for binding viral RNA, the C-terminal catalytic domain is also involved in regulation as GAPC fragments with no catalytic domain may still bind negative-strand RNA, but repress rather than stimulate viral replication (Huang and Nagy, 2011). Since the catalytic cysteine of GAPC is close to its putative RNA-binding domains, the capability of GAPC to affect viral replication is likely, though not demonstrated, to be redox regulated.

Moonlighting properties of GAPC, possibly shared with other glycolytic enzymes, are also suggested by the specific and dynamic binding to sub-cellular structures such as membranes and the cytoskeleton. A portion of all glycolytic enzymes, including GAPC, is associated to the cytoplasmic face of the outer membrane of plant mitochondria (Giegé et al., 2003). Binding of glycolytic enzymes to the organelle is dynamic and favored by conditions of high respiratory demands. Organelle-bound glycolytic enzymes may support respiration by directly providing pyruvate to mitochondria and restricting the use of glycolytic intermediates by alternative metabolic pathways (Graham et al., 2007). However, mitochondrial association of Arabidopsis GAPCs was also increased by H_2_O_2_- treatments (Sweetlove et al., 2002), i.e., under conditions in which GAPDH activity may be inhibited. The interaction between GAPCs and mitochondria is probably mediated by voltage-dependent anion channels (VDAC; Graham et al., 2007; **Figure 9**), and *in vitro* the binding of GAPC1 to VDAC3 was actually found to be inhibited by DTT (Wojtera-Kwiczor et al., 2013). Under oxidative stress conditions, it is thus possible that GAPC, and perhaps other glycolytic enzymes like aldolase, are induced to associate to mitochondria in an oxidized and inactive state that may play an alternative function to respiration. In mammals, binding of GAPDH to VDAC was also found to be inhibited by DTT and suggested to induce mitochondrial membrane permeabilization and apoptosis (Tarze et al., 2007). Whether a similar role might also be played by plant GAPC remains to be investigated in detail.

Plant glycolytic GAPDH can also interact with the cytoskeleton and promote actin bundling, again a function of GAPDH that is stimulated by oxidizing conditions (Wojtera-Kwiczor et al., 2013). GSNO causes both glutathionylation and S-nitrosylation of GapC (Zaffagnini et al., 2013) and GSNO-treated GAPC1 binds F-actin in a DTT-reversible manner (Wojtera-Kwiczor et al., 2013; **Figure 9**). Generally speaking, the complex relationships between the cellular redox state, the GAPDH redox state and enzyme activity, and its subcellular localization (cytoplasm, nucleus, plasma membrane, mitochondria, cytoskeleton) speaks in favor of a possible role as an oxidative stress sensor, but clearly our comprehension of the system is still fragmental.

## CONCLUSION

Glyceraldehyde-3-phosphate dehydrogenase is an abundant protein of animal and plant cells, whose catalytic role in glycolysis is based on a highly reactive catalytic cysteine. GAPDH is also a moonlighting, multifunctional protein with non-glycolytic roles that often involve oxidative modifications of the catalytic cysteine. Plant cytoplasmic GAPDH (GAPC) proved an excellent tool to study the different types of redox PTMs that may affect a plant protein (Holtgrefe et al., 2008; Guo et al., 2012; Bedhomme et al., 2012; Piattoni et al., 2013; Zaffagnini et al., 2013), and it is now established that also GAPC, like animal GAPDH, can be translocated to the nucleus under particular, oxidizing conditions (Waver et al., 2010; Kim et al., 2013; Vescovi et al., 2013). However, neither the mechanism of translocation nor the function of GAPC in the plant nucleus are currently understood. In spite of its popularity, the role of GAPC in plant cell death is still speculative, whereas it is well established that the nuclear translocation of S-nitrosylated GAPDH can transmit an apoptotic stimulus in animal cells (Hara et al., 2005; Sen et al., 2009; Kornberg et al., 2010; Nakamura and Lipton, 2013). Nonetheless, plant GAPC is a moonlighting protein and other non-glycolytic roles have been recently established, including the control of transpiration in Arabidopsis plants (via ROS-dependent activation of phospholipase D-δ; Guo et al., 2012), and the regulation of the replication of phytopathogenic viruses (via binding of viral RNA; Prasanth et al., 2011). Interestingly both functions are, or likely to be, redox-regulated and thus affected by perturbations of the cellular redox homeostasis.

Every function of GAPDH that depends, or is regulated by a redox modification that blocks its catalytic activity is by definition alternative to its role in glycolysis. However, alternative functions of GAPC, signaling functions in particular, may well involve only a small fraction of the large pool of GAPC in plant cells. Moreover, plant cells contain metabolic alternatives to keep the glycolytic flux operative under conditions in which GAPC is oxidatively inactivated. Overall, GAPC appears to have suitable properties to behave as an oxidative stress sensor in plant cells and this function may not be in contrast with its classical glycolytic role.

## Conflict of Interest Statement

The authors declare that the research was conducted in the absence of any commercial or financial relationships that could be construed as a potential conflict of interest.
